# Development and validation of a risk prediction model of prehospital delay in patients with acute ischemic stroke

**DOI:** 10.3389/fpubh.2026.1737563

**Published:** 2026-02-11

**Authors:** Yuzhen Bai, Mingjing Zhou, Xueyan Luo, Yu Zhong

**Affiliations:** Department of Neurology, The People’s Hospital of Yubei District of Chongqing City, Chongqing, China

**Keywords:** acute ischemic stroke, build and validate, nomogram, predictive model, prehospital delay

## Abstract

**Background:**

Prehospital delay is the primary cause of low reperfusion treatment rates among patients with acute ischemic stroke (AIS). Healthcare providers lack tools to identify high-risk patients, and thus predictive models need to be developed to screen high-risk populations, aiming to reduce the incidence of delayed medical care among AIS patients.

**Objectives:**

This study was conducted to investigate the factors influencing delayed medical care among AIS patients and construct and validate a predictive model for the risk of prehospital delay.

**Methods:**

By conducting convenience sampling, 348 AIS patients admitted to the neurology department of a tertiary hospital in China between September 2024 and June 2025 were enrolled. The patients were divided into a prehospital delay group and Non-prehospital delay group based on whether the time from the onset of symptoms to hospital admission exceeded 4.5 h (the critical time window for reperfusion therapy). Univariate and logistic regression analyses were performed to identify factors influencing delayed medical care. Using the R software, a risk prediction model incorporating a delay-to-treat line chart was constructed for AIS patients, followed by internal validation.

**Results:**

Among 348 AIS patients, 281 experienced prehospital delay, resulting in a delay rate of 80.8%. Logistic regression analysis identified education, History of cerebral infarction, place of onset, Subsequent measures, and National Institutes of Health Stroke Scale (NIHSS) score as independent risk factors. The Hosmer–Lemeshow test yielded a *χ*^2^ value of 9.84 (*p =* 0.277). The area under the ROC curve (AUC) of the model was 0.87, with an optimal cutoff value of 0.625, sensitivity of 0.79, and specificity of 0.84. The calibration curves demonstrated good agreement between the predicted and actual incidence rates, whereas the decision curves confirmed the clinical net benefit of the model.

**Discussion:**

The constructed risk prediction model shows strong predictive efficacy and can help clinical nurses rapidly identify high-risk individuals for prehospital delay in AIS patients and implement targeted interventions. This approach improves healthcare-seeking behavior among patients and decreases the risk of prehospital delay.

## 1 Introduction

Acute ischemic stroke (AIS) accounts for 69.6–72.8% of all types of stroke, and it is a very common brain disease around the world (1). Effective treatment of AIS involves the use of recombinant plasminogen activator (rt-PA) 4.5 h after symptoms or 4.5–6 h after illness (2). In the early stages of the disease, reperfusion-based interventions, such as intravenous thrombolysis and mechanical thrombectomy, can restore patency to occluded vessels, reestablish blood flow to ischemic brain tissue, and thereby promote early recovery of neurological function (3). However, both intravenous thrombolysis and mechanical embolization are critical time interventions, and each intervention has a fixed treatment time window (4). Therefore, adequate treatment needs to be administered within this window for a favorable patient prognosis.

Patients with AIS who are unable to access a medical facility equipped to provide stroke-specific care within the narrow therapeutic window miss the chance to receive effective intervention (5). This delay adversely affects treatment outcomes and long-term prognosis, potentially endangering the lives of the patients. Delayed presentation is prevalent not only in large general hospitals in China but also in specialized stroke centers across Europe and America (6). Reports indicate that the rate of central venous thrombolysis in Europe and the United States is not high, ranging from 4.1 to 6.3% (7). According to a stroke report from the United States, about 75.0% of patients took longer than the recommended time window of 120 min to be transferred from their initial hospital to a comprehensive stroke center, and the median transfer delay was found to be 174 min (8). For some patients, transport delays result in the administration of critical treatments, such as mechanical thrombectomy, more than 3 h after the onset of symptoms (9). A European study revealed that only 25.0% of stroke patients gained hospital admission within the time window of 3.5 h after the onset of symptoms, whereas half of the patients did not reach the hospital for treatment until more than 14 h after onset (10). Another study indicated that 68.6% of patients reached the hospital within 4 h of the onset of symptoms, among whom 63.2% received intravenous thrombolysis, whereas 36.8% did not receive this treatment (11). A UK stroke report indicated a median prehospital delay of 4.7 h for AIS patients, with 39.5% admitted for treatment only after 3 h of the onset of symptoms (12). In Australia, only 41.0% of AIS patients received treatment within 4.5 h (13). In China, only 1.6–1.9% of stroke patients undergo intravenous thrombolysis, which is significantly lower than the rates reported in developed countries (14).

Many studies have identified a range of factors associated with prehospital delay in patients with AIS. Research indicates that the distance from the hospital at onset, mode of transport, distance to the first-contact hospital, and whether emergency services are used after the onset of symptoms all influence treatment delays in AIS patients (15). A study in Indonesia revealed that delayed medical assistance after symptom onset, socioeconomic conditions, lack of recognition of stroke symptoms, and transportation difficulties all hinder timely treatment (16). Additional studies have shown that factors such as the education level of patients, place of residence, disease severity, and knowledge of stroke are similarly associated with prehospital delay in AIS patients (17).

Prehospital delay in patients with AIS is a globally prevalent issue, highlighting the need to ensure that patients have access to effective treatment within the designated therapeutic window. Many studies have comprehensively investigated prehospital delay among patients with breast cancer, lung cancer, and various chronic diseases. Therefore, the rapid and accurate identification of symptoms, along with the implementation of corresponding preventive measures, is necessary (18, 19). Risk prediction models serve as effective tools for identifying prehospital delay among AIS patients, allowing nursing staff to quickly identify high-risk individuals and thereby minimize the occurrence of delayed care. In this study, we identified the factors influencing prehospital delay in patients with AIS and developed a risk prediction model tailored to these patients. This study may provide nurses with a more user-friendly risk assessment tool and act as a basis for formulating targeted prevention and intervention strategies.

## 2 Methods

### 2.1 Study design and sample

We adopted a prospective study design with primary data collection. From September 2024 to June 2025, a convenience sampling method was used to recruit and survey patients with AIS admitted to the Comprehensive Treatment Center for Neurological Diseases at a tertiary hospital in China.

#### 2.1.1 Study setting

This study was conducted at the Comprehensive Treatment Center for Neurological Diseases in a Grade III Class A general hospital in Yubei District, Chongqing, Southwest China. The hospital is certified as a Regional Key Stroke Center by the local health administrative department, with a specialized neurology team focusing on acute cerebrovascular disease management. The department operates 100 inpatient beds, implements a standardized stroke care pathway (including rapid emergency triage, neuroimaging diagnosis, and reperfusion therapy), and admits about 3,000 AIS patients annually. All participants were recruited from this department, ensuring consistent clinical management protocols and data collection standards throughout the study period.

#### 2.1.2 Selection of participants

The inclusion criteria for patients were as follows: ① diagnosis according to the criteria specified in the 2023 Chinese Guidelines for the Diagnosis and Treatment of Acute Ischemic Stroke, diagnosed with AIS by cranial CT or MRI (20); ② age ≥18 years; ③ time from symptom onset to presentation ≤14 days (21); ④ ability to communicate and understand the content of the questionnaire; and ⑤ provision of informed consent and voluntary participation. The exclusion criteria were as follows: ① patients whose exact time of onset was unclear; ② patients with concomitant malignant tumors, end-stage renal disease, major organ transplantation, or other severe illnesses; and ③ patients with mental or cognitive disorders or inability to communicate.

#### 2.1.3 Sample size

The sample size was estimated using the standard formula for calculating prospective observational study sample size, which is widely applied in clinical studies that aim to identify risk factors for a binary outcome. This formula is based on the Guidelines for Clinical Research Sample Size Calculation, ensuring methodological standardization (22). The size calculation formula is as follows:


$$n=[Z_{\alpha /2}\pi (1-\pi )]/\delta^{2}$$

The parameters are set as follows: In this formula, the sample size is n, the test statistic is Z, π indicates the population proportion, δ indicates the margin of error, *α* is set to 0.05 for a two-tailed test, and the confidence interval (CI) is set to 95%. When α is 0.05, Z_α/2_ = 1.96. Research has shown that the rate of delayed medical treatment for AIS is 71% (23). *π* = 0.71, δ = 0.05.

Substituting Z_α/2_ = 1.96, *π* = 0.71, δ = 0.05 yields n=316, plus 10% dropout gives 348 cases.

### 2.2 Ethics considerations

This study was conducted according to the Declaration of Helsinki. The study protocol was reviewed and approved by the Institutional Ethics Committee of the participating tertiary hospital (Approval No: 2024SA21). All participants provided informed consent before data collection and could withdraw at any time.

### 2.3 Instruments

#### 2.3.1 General information questionnaire

The research team ascertained the following study variables: age, gender, education, marital status, monthly Income, past medical history, and place of onset. Disease-related information, including place of onset, first hospital visit, and mode of transportation, was collected. The following indicators were checked: patient blood pressure, low-density lipoprotein cholesterol, and triglycerides. According to the time window for intravenous thrombolysis and related literature, if the duration from symptom onset to hospital admission for treatment exceeds 4.5 h, it is defined as prehospital delay; if this time is within 4.5 h, it is regarded as non-prehospital delay (24).

#### 2.3.2 National Institutes of Health Stroke Scale (NIHSS)

The scale is used to evaluate nerve deficits, disease progression, and recovery in patients with acute stroke (25). The higher the NIHSS score, the greater the degree of damage related to stroke. The scale consists of 13 elements, including the score of each element. The score ranges from 0 to 42. The higher the score, the greater the degree of nerve damage. The NIHSS score was categorized into four grades based on standard clinical criteria: mild (0–4 points), moderate (5–15 points), moderate–severe (16–20 points), and severe (21–42 points).

#### 2.3.3 Perceived Social Support Scale (PSSS)

This scale, developed by Zimet, is primarily used to assess the self-perceived social support of individuals (26). This tool was subsequently adapted for Chinese readers by Sacks et al. (27). It includes three dimensions, including peer support, family support, and other people’s support, with a total of 12 items. Lickert’s seven-point scoring system ranges from 1 to 7, corresponding to the range of “strongly disagree” to “disagree.” The total score ranges from 12 to 84; 12–36: low level of support; 37–60: medium level of support; 61–84: high level of support. The higher the total score of the scale, the greater the corresponding perceived level of social support. The *α* coefficient of the Krombach scale is 0.840, which has good reliability and validity and is extensively used for patients with stroke, diabetes, lung cancer, and other diseases.

#### 2.3.4 Perceived Barriers to Medical Attendance Behavior Scale

The Perceived Barriers to Medical Care Behavior Scale was developed by Al-Hassan and Omran (28). Researchers have used it to evaluate the level of perceived obstacles in medical decision-making, including identifying the severity of diseases and identifying aspects of medical obstacles. The Chinese adaptation of this scale, localized by Li et al. (29), comprises 10 items, with the items as follows: traveling to the hospital requires significant time, preferring to tolerate pain instead of troubling the doctor, feeling embarrassed to visit the hospital immediately after symptom onset, finding hospital visits uncomfortable, perceiving disease diagnosis and treatment as painful, putting pressure on the family, limiting the social activities of the affected individual, hindering work completion, struggling to give up hobbies and habits, and needing to consult family members before going to the hospital. The rating range of each scale element is 1 to 6 points, ranging from “very inconsistent” to “accurate consistent”; the higher the rating is between 10 and 60 points, the greater the perceptibility of medical behavior, and the alpha ratio is 0.74.

### 2.4 Data collection methods

Before data collection, all research team members underwent standardized training. After the training was completed, clinical data collection began. Research participants were rigorously selected based on the study objectives. Patients were informed of the study purpose, procedures, and rights before data collection. All patients provided written informed consent. Researchers collected data using paper questionnaires designed for the study objectives. During patient visits, one-on-one administration of the paper questionnaires with standardized instructions was conducted in the ward, and patient responses were recorded. All completed paper questionnaires were subjected to double-entry data verification by two independent researchers before being electronically input. Each questionnaire was individually reviewed for completeness and accuracy. The data were entered into Microsoft Excel 2019 by two researchers, followed by a final cross-check to ensure data authenticity and reliability.

To ensure that data management was rigorous, a series of quality control measures was implemented during the electronic data entry and storage process. Specific quality control measures included the following: ① Data entry performed by two independent researchers, with discrepancies resolved by consulting original paper questionnaires; ② Ensuring standardized entry to guarantee consistency, such as decimal places, thousand separators, numerical or percentage formats, and even alignment; ③ Access control: Electronic datasets were stored on password-protected hospital servers. Editing privileges were restricted to designated research team members, whereas supervisors had read-only access to prevent data tampering or modification. ④ All data modifications were tracked using the “Track Changes” feature in Excel, recording the editor, revision time, and details to ensure full traceability of alterations. These measures collectively protected the integrity and reliability of the data.

### 2.5 Study variables

#### 2.5.1 Outcome variable

The primary outcome variable of this study was prehospital delay in AIS patients, defined as the time interval from the onset of stroke symptoms (e.g., limb weakness, slurred speech) to the arrival of the patient at the hospital emergency department exceeding 4.5 h; this cutoff value was determined based on the time window for intravenous thrombolysis (the core reperfusion therapy for AIS) recommended by the Chinese Guidelines for the Diagnosis and Treatment of Acute Ischemic Stroke (2023 Edition). Patients were categorized into two groups: the prehospital delay group (symptom-to-hospital time > 4.5 h) and the non-prehospital delay group (symptom-to-hospital time ≤ 4.5 h).

#### 2.5.2 Predictor variables

Based on a literature review and clinical relevance, a comprehensive set of potential predictors was collected. These were organized into the following domains:

Sociodemographic factors: Age, gender, education, place of residence, monthly income, and marital status. Clinical characteristics and history: hypertension, diabetes, History of cerebral infarction, History of atrial fibrillation, smoking, and alcohol consumption. Clinical measures included the NIHSS, systolic blood pressure, diastolic blood pressure, and low-density lipoprotein cholesterol. The stroke event characteristics included the place of onset(At home/workplace/outdoors), Individuals with detected symptoms, Subsequent measures (call emergency services/Immediately contact family or friends to go to the hospital/Wait and monitor symptom changes), the first medical facility contacted, the mode of transportation to the hospital, and the distance to the first hospital. Scale: PSSS score and perceived barriers to seeking healthcare (PBHSD-C score).

### 2.6 Statistical methods

The data were analyzed using SPSS 26.0 statistical software. Continuous variables were first tested for normality using the Shapiro–Wilk test. Variables that followed a normal distribution were reported as the mean ± standard deviation. The differences were determined by conducting the independent samples t-test (between two groups) or one-way analysis of variance (ANOVA) (among multiple groups). Non-normally distributed continuous variables (e.g., NIHSS score and time from symptom onset to admission) were reported as medians and interquartile ranges [M (Q1, Q3)]. The differences were determined by conducting the Mann–Whitney U test (between two groups) or the Kruskal-Wallis H test (among multiple groups). Categorical variables were presented as frequencies and percentages [*n* (%)], and the data were compared using the *χ*^2^ test or Fisher’s exact test (for the expected frequency <5). Univariate analysis was performed to initially screen potential predictors of prehospital delay. For continuous predictors (e.g., patient age and the NIHSS score), univariate logistic regression was performed to assess their associations with prehospital delay, and the results were reported as odds ratios (ORs) with 95% confidence intervals (CIs). For categorical predictors (e.g., education, history of cerebral infarction, place of onset, and first measures after onset), univariate logistic regression was also conducted after dummy variable coding, with ORs and 95% CIs reported to quantify the strength of association.

Multivariate binary logistic regression (enter method) was then performed to identify independent predictors for prehospital delay. All variables with *p* < 0.10 from the univariate analysis were entered into the initial model. The outcome was coded as 1 for prehospital delay (symptom-to-hospital time >4.5 h) and 0 for non-prehospital delay (symptom-to-hospital time ≤4.5 h). The results are presented as adjusted ORs with 95% CIs. A risk prediction model using a nomogram was constructed using the R 4.5.1 software and the loaded rms package.

The specific methods for constructing the nomograms were as follows: first, the unstandardized regression coefficients (*β*) of all independent predictors in the final logistic model were extracted; second, these β coefficients were scaled to a common range to facilitate graphical representation of a unified point system; third, the scaled coefficients were converted into a user-friendly point scale for each predictor level, with the reference category set at 0 points; and finally, the total point score (sum of individual predictor points) was mapped to the predicted probability of prehospital delay using the inverse logistic function. Then, the nomogram was visualized with axes for each predictor, a total point axis, and a predicted probability axis.

Internal validation of the prediction model was performed using the bootstrap resampling method with 1,000 repetitions. The procedure involved repeatedly drawing random samples with replacement from the original dataset to create multiple bootstrap samples of the same size. The model was rebuilt on each bootstrap sample and then tested on the original sample to estimate its optimism, i.e., overfitting. Model performance was evaluated based on three key metrics: the concordance index (C-index) for discrimination, the calibration curve for calibration, and decision curve analysis (DCA) for clinical utility.

The calibration of a model serves as a key indicator for evaluating the accuracy of a disease risk model while predicting the probability of an individual experiencing an outcome event in the future. It reflects the degree of consistency between the risk predicted by the model and the actual occurrence of risk. The Hosmer–Lemeshow test was conducted to validate the model’s goodness of fit.

The discriminatory ability of a model is assessed by plotting a receiver operating characteristic (ROC) curve and evaluating the AUC. A larger AUC indicates better predictive discrimination capability. Generally, an AUC < 0.6 indicates poor discriminatory ability, 0.6–0.75 indicates moderate discriminatory ability, and >0.75 indicates good discriminatory ability.

Decision curve analysis (DCA) was conducted to evaluate the clinical utility of the predictive model. By comparing the model against the “All” (complete treatment) and “None” (no treatment) reference strategies, the DCA visually illustrates the net benefit of the model across different threshold probabilities. If the model curve consistently demonstrates a net benefit superior to both reference strategies within a specific threshold probability range, it indicates that using the model to guide clinical decisions within that range yields greater net benefit and holds practical clinical value. In contrast, if the model curve approaches the reference strategy curves within a certain threshold range (showing no significant net benefit advantage), it suggests limited clinical utility for the model in that range. This outcome provides a direct reference for clinicians in the formulation of individualized intervention strategies. Model predictive performance was determined to confirm sensitivity, specificity, and accuracy, with *p* < 0.05 indicating statistically significant differences.

## 3 Results

### 3.1 General information of the participants

A total of 348 inpatients were included in this study, including 231 males (66.4%) and 117 females (33.6%), with an age of 68.1 ± 12.2 years. Among all participants, 231 participants (66.4%) received primarily school-level education or below. There were 240 urban residents (69.0%) and 94 rural residents (27.0%). There were 137 participants (39.4%) with a history of smoking and 112 participants (32.2%) with a history of alcohol consumption. There were 254 participants (73.0%) with a history of hypertension. A total of 258 patients (74.1%) themselves discovered the initial symptoms of the disease. There were 277 cases (79.6%) in which the individuals were not aware that a stroke had occurred after the onset of symptoms. A total of 115 patients (33.0%) had never undergone a physical examination before, whereas 149 patients (42.8%) first chose to wait and observe their symptoms after the onset of illness.

### 3.2 Prehospital delay-seeking behavior in patients with acute ischemic stroke

Among the 348 hospitalized patients, 281 had prehospital delay, leading to an incidence rate of prehospital delay of 80.8%. The duration from patients’ self-perceived onset of illness to obtaining effective treatment at a medical institution was 33.7 ± 3.0 h, and the median duration was 24 h. Among these patients, 10 patients (2.9%) arrived at the hospital within 4.5–6 h, 134 patients (38.5%) arrived within 6–24 h, and 136 patients (39.1%) arrived after more than 24 h.

### 3.3 Univariate analysis of prehospital delay among patients with AIS

Univariate analysis of the prompt care group and delayed care group revealed that educational attainment, place of residence, monthly income, history of cerebral infarction, history of atrial fibrillation, Place of onset, initial medical facility, awareness of stroke onset after symptom onset, Subsequent measures, transport, distance from initial hospital, NIHSS score, PSSS score, and PBHSD-C score were factors influencing prehospital delay seeking among patients with acute ischemic stroke. Specific details regarding univariate variance information for patients with AIS experiencing prehospital delay are presented in Table 1.

**Table 1:** Baseline characteristics and univariate analysis of prehospital delay among AIS patients (*n* = 348).

Variables	Total (*n* = 348)	Prehospital delay group (*n* = 281)	Non-prehospital delay group (*n* = 67)	*p* values
Gender, *n* (%)				0.891
Male	231 (66.4)	187 (66.5)	44 (65.7)	
Female	117 (33.6)	94 (33.5)	23 (34.3)	
Age, M [Q1, Q3] (years)	70 [59, 77]	70 [60, 77]	67 [58, 78]	0.616
Education				<0.001^***^
Elementary school or below	213 (61.2)	185 (65.8)	28 (41.8)	
Junior High	92 (26.4)	73 (26.0)	19 (28.4)	
High School/Vocational School	29 (8.3)	15 (5.3)	14 (20.9)	
College degree and above	14 (4.0)	8 (2.8)	6 (9.0)	
Marital Status				0.441
Married	274 (78.7)	223 (79.4)	51 (76.1)	
Unmarried	7 (2.0)	6 (2.1)	1 (1.5)	
Divorced	4 (1.1)	2 (0.7)	2 (3.0)	
Widowed	63 (18.1)	50 (17.8)	13 (19.4)	
Place of residence				0.013^*^
Rural	94 (27.0)	84 (29.9)	10 (14.9)	
Urban	254 (73.0)	197 (70.1)	57 (85.1)	
Current Living Situation				0.688
Living Alone	54 (15.5)	43 (15.3)	11 (16.4)	
Living with a spouse	212 (60.9)	168 (59.8)	44 (65.7)	
Living with children	55 (15.8)	47 (16.7)	8 (11.9)	
Living with children and a spouse	27 (7.8)	23 (8.2)	4 (6.0)	
Monthly Income				0.008^**^
Below 3,000 yuan	118 (33.9)	101 (35.9)	17 (25.4)	
3,001–4,000 yuan	94 (27.0)	82 (29.2)	12 (17.9)	
4,001–5,000 yuan	83 (23.9)	62 (22.1)	21 (31.3)	
>5,000 yuan	53 (15.2)	36 (25.4)	17 (25.4)	
Smoking				0.702
Yes	137 (39.4)	112 (39.9)	25 (37.3)	
No	211 (60.6)	169 (60.1)	42 (62.7)	
Alcohol consumption				0.766
Yes	109 (31.3)	87 (31.0)	22 (32.8)	
No	239 (68.7)	194 (69.0)	45 (67.2)	
Diabetes				0.315
None	253 (72.7)	201 (71.5)	52 (77.6)	
Yes	95 (27.3)	80 (28.5)	15 (22.4)	
Hypertension				0.976
None	94 (27.0)	76 (27.0)	18 (26.9)	
Yes	254 (73.0)	205 (73.0)	49 (73.1)	
History of cerebral infarction				0.002^**^
None	304 (87.4)	253 (90.0)	51 (76.1)	
Yes	44 (12.6)	28 (10.0)	16 (23.9)	
History of atrial fibrillation				0.041^*^
None	310 (89.1)	255 (90.7)	55 (82.1)	
Yes	38 (10.9)	26 (9.3)	12 (17.9)	
Physical Examination Status				0.825
Several times a year	19 (5.5)	16 (5.7)	3 (4.5)	
Once a year	149 (42.8)	117 (41.6)	32 (47.8)	
Every few years	65 (18.7)	53 (18.9)	12 (17.9)	
Never undergo physical examinations	115 (33.0)	95 (33.8)	20 (29.9)	
Place of onset				0.006^**^
At home	264 (75.9)	223 (79.4)	41 (61.2)	
Workplace	19 (5.5)	12 (4.3)	7 (10.4)	
Outdoors	65 (18.7)	46 (16.4)	19 (28.4)	
Initial medical facility				0.006^**^
Community/Township Health Center	51 (14.7)	49 (17.4)	2 (3.0)	
County-level hospitals	34 (9.8)	29 (10.3)	5 (7.5)	
Tertiary General Hospitals	263 (75.6)	203 (72.2)	60 (89.6)	
Individuals with detected symptoms				0.112
Self	258 (74.1)	210 (74.7)	48 (71.6)	
Relatives	72 (20.7)	60 (21.4)	12 (17.9)	
Colleagues	8 (2.3)	4 (1.4)	4 (6.0)	
Stranger	10 (2.9)	7 (2.5)	3 (4.5)	
Awareness of stroke onset after symptom onset				<0.001^***^
Aware	71 (20.4)	46 (16.4)	25 (37.3)	
Unaware	277 (79.6)	235 (83.6)	42 (62.7)	
Subsequent measures				<0.001^***^
Calling emergency services (120)	49 (14.1)	21 (7.5)	28 (41.8)	
Immediately contact family or friends to go to the hospital	150 (43.1)	115 (40.9)	35 (52.2)	
Wait and monitor symptom changes	149 (42.8)	145 (51.6)	4 (6.0)	
Transportation				<0.001^***^
Ambulance	53 (15.2)	23 (8.2)	30 (44.8)	
Private vehicle	196 (56.3)	169 (60.1)	27 (40.3)	
Public Transportation	41 (11.8)	39 (13.9)	2 (3.0)	
Walking	9 (2.6)	6 (2.1)	3 (4.5)	
Taxi	49 (14.1)	44 (15.7)	5 (7.5)	
Distance from initial hospital/km				0.020^*^
<5 km	165 (47.4)	128 (45.6)	37 (55.2)	
5–10 km	66 (19.0)	49 (17.4)	17 (25.4)	
>10 km	117 (33.6)	104 (37.0)	13 (19.4)	
Systolic Blood Pressure	146.7 ± 19.2	146.0 ± 18.9	149.5 ± 20.2	0.172
Diastolic Blood Pressure (mmHg)	86.9 ± 14.0	86.9 ± 13.9	86.9 ± 14.4	0.947
LDL cholesterol (mmol/L)	2.7 ± 0.8	2.7 ± 0.8	2.9 ± 0.9	0.661
NIHSS, M [Q1, Q3]	2 [1, 3]	1 [1, 3]	2 [1, 5]	0.004^**^
PSSS, mean ± SD	45.3 ± 9.8	44.8 ± 9.4	47.6 ± 11.0	0.038^*^
PBHSD-C	38.9 ± 17.1	37.9 ± 18.5	43.3 ± 7.1	0.018^*^

PSSS represents the Perceived Social Support Scale score; PBHSD-C denotes the Perceived Barriers to Healthcare Scale (Chinese version) score. Categorical data are presented as *n* (%). Continuous variables are reported as the mean ±SD when normally distributed and reported as the median [Q1, Q3] when non-normally distributed. Normally distributed continuous variables were analyzed by conducting the independent samples t-test, non-normally distributed continuous variables were analyzed by conducting the Mann–Whitney U test, and categorical variables were analyzed by conducting the *χ*^2^ test or Fisher’s exact test (when the expected frequency was <5); *p < 0.05, **p < 0.01, and ***p < 0.001.

### 3.4 Logistic regression analysis of prehospital delay for AIS

Statistically significant factors from univariate analysis were used as independent variables, and binary logistic regression was performed, where prehospital delay (0 = no delay, 1 = delay) served as the dependent variable. The coding details are presented in the Supplementary material. The results of the binary logistic regression are presented in Table 2. Education level (specifically, high school/vocational school level), a history of cerebral infarction, subsequent measures, and the NIHSS score were identified as independent predictors of prehospital delay (all *p* < 0.05). The variable “place of onset” was retained in the final model because of its *a priori* clinical relevance for prehospital delay, although it did not reach statistical significance (*p* = 0.100), as detailed in Table 2.

**Table 2:** Binary logistic regression analysis of prehospital delay for AIS (*n* = 348).

Variable	*B*	*SE*	*p*-values	*OR*	95% *CI*
Education (Reference: Elementary school or below)	–	–	0.024	–	–
Junior High	−0.310	0.387	0.422	0.733	0.344–1.564
High school/vocational school	−1.458	0.517	0.005	0.233	0.084–0.641
Bachelor’s degree or higher	−1.191	0.738	0.107	0.304	0.072–1.292
History of cerebral infarction	−0.908	0.423	0.020	0.373	0.163–0.856
Place of onset (Reference: At home)	-	-	0.100	-	-
Workplace	−0.895	0.596	0.133	0.409	0.127–1.314
Outdoors	−0.732	0.412	0.076	0.481	0.214–1.079
Subsequent measures (Reference: Calling emergency services 120)	–	–	<0.001	–	–
Immediately contacted family/friends to go to hospital	1.339	0.386	0.001	3.816	1.791–8.131
Waited and observed symptom changes	3.523	0.610	< 0.001	33.896	10.245–112.144
NIHSS	−0.119	0.040	0.003	0.888	0.821–0.961
Constant	0.970	0.420	0.021	2.639	–

① Reference groups for categorical variables were as follows: Educational attainment, “Elementary school or below”; place of onset, “At home”; first measures taken after onset, “Calling emergency services 120.” ② A *p*-value < 0.05 was considered to be statistically significant. ③ Abbreviations: NIHSS, National Institutes of Health Stroke Scale; OR, odds ratio; CI, confidence interval. ④ The variable ‘place of onset’ was retained in the final model due to its clinical relevance, despite an overall *p* = 0.100.

### 3.5 Development and internal validation of a risk prediction model for prehospital delay in AIS

**Figure 1:**
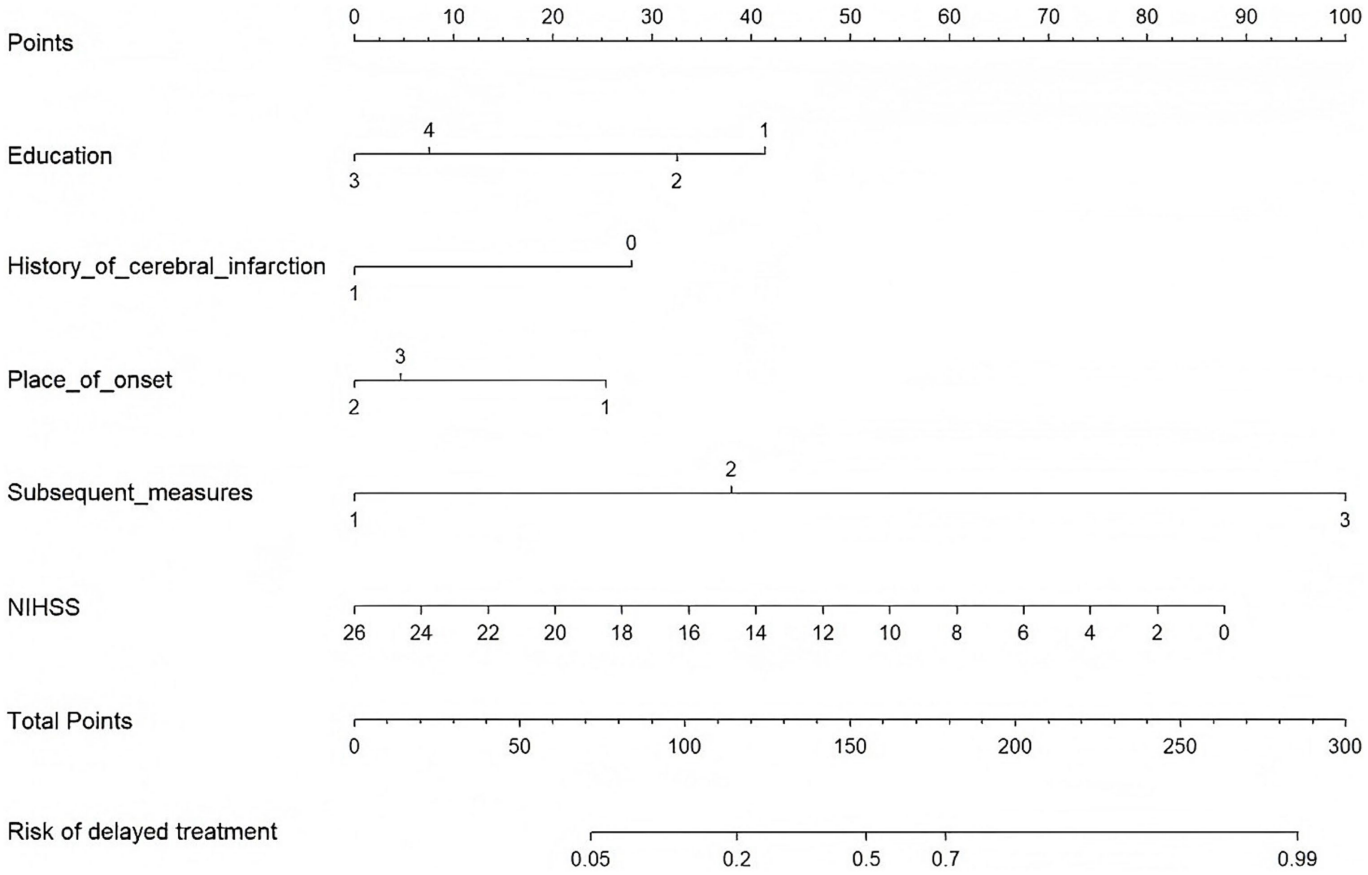
Nomogram for predicting the risk of prehospital delays in AIS patients.

**Figure 2:**
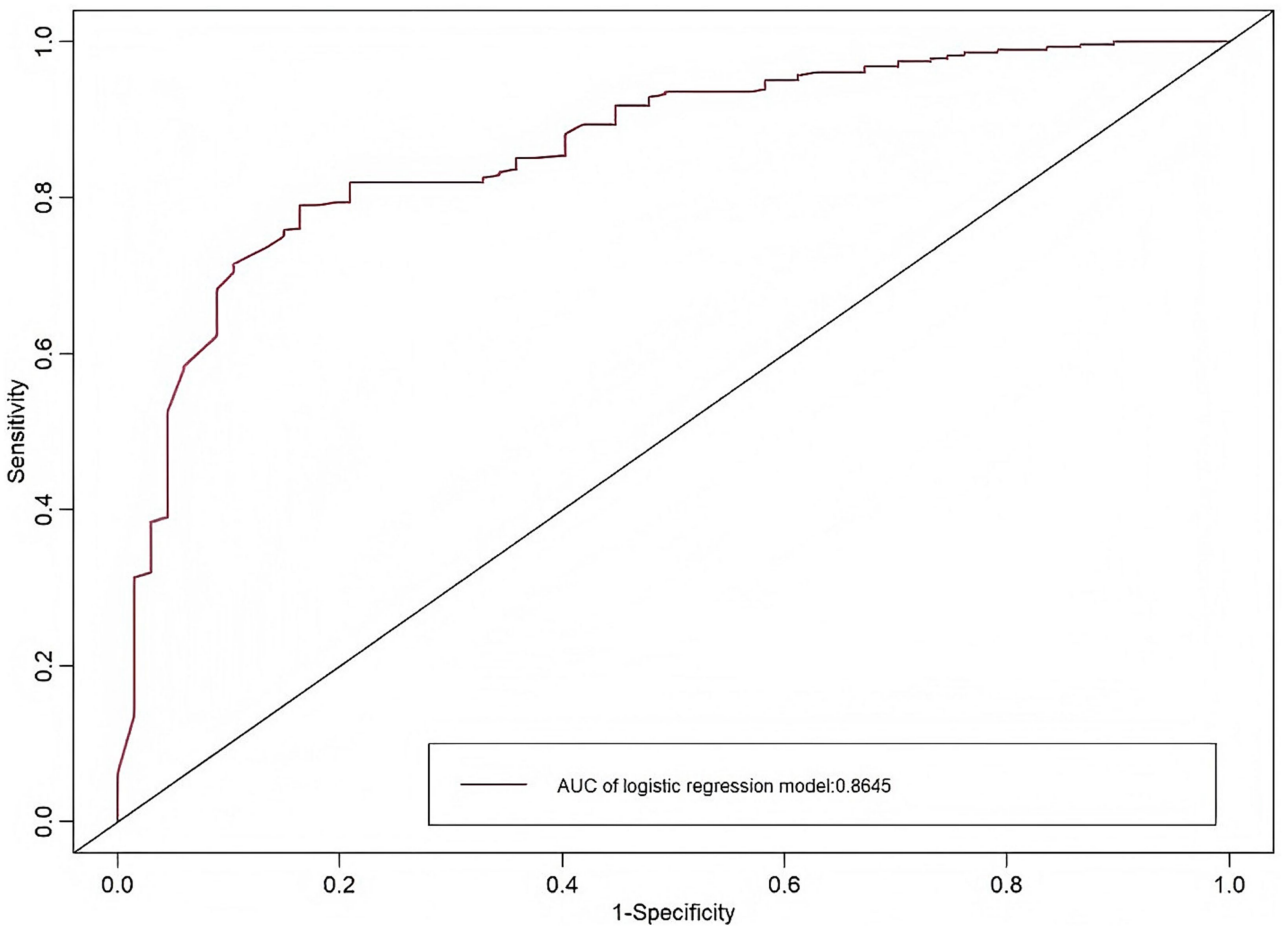
ROC curve of the risk classification model for prehospital delay in AIS patients.

Based on the results of binary logistic regression analysis, a predictive model for the risk of prehospital delay in AIS patients was established using educational attainment, history of cerebral infarction, location of onset, initial measures taken after onset, and the NIHSS score as predictors. The prediction formula was as follows: Logit(P) = 0.97–0.31 × Junior high school−1.46 × High school/vocational school−1.19 × Bachelor’s degree or higher−0.91 × History of Cerebral Infarction−0.90 × Workplace−0.73 × Outdoors+1.34 × Immediately contacted family/friends to go to hospital+3.52 × Waited and observed symptom changes-0.12 × NIHSS score. A scorer plot is illustrated in Figure 1. Each predictor in the figure corresponds to a score on the upper scale. By summing all the factors, the total score was derived. The vertical line extending downward from the total score axis reflected the predicted probability of delayed presentation in AIS patients. Internal validation through 100 bootstrap resamples revealed that the optimal cutoff value was 0.625, with a sensitivity of 0.79 and a specificity of 0.84, suggesting that the model was well-fitted. The area under the ROC curve was 0.87 (95% *CI*, 0.82–0.91), which indicated that the constructed predictive model for prehospital delay in AIS patients possessed clinical diagnostic significance, as illustrated in Figure 2.

**Figure 3:**
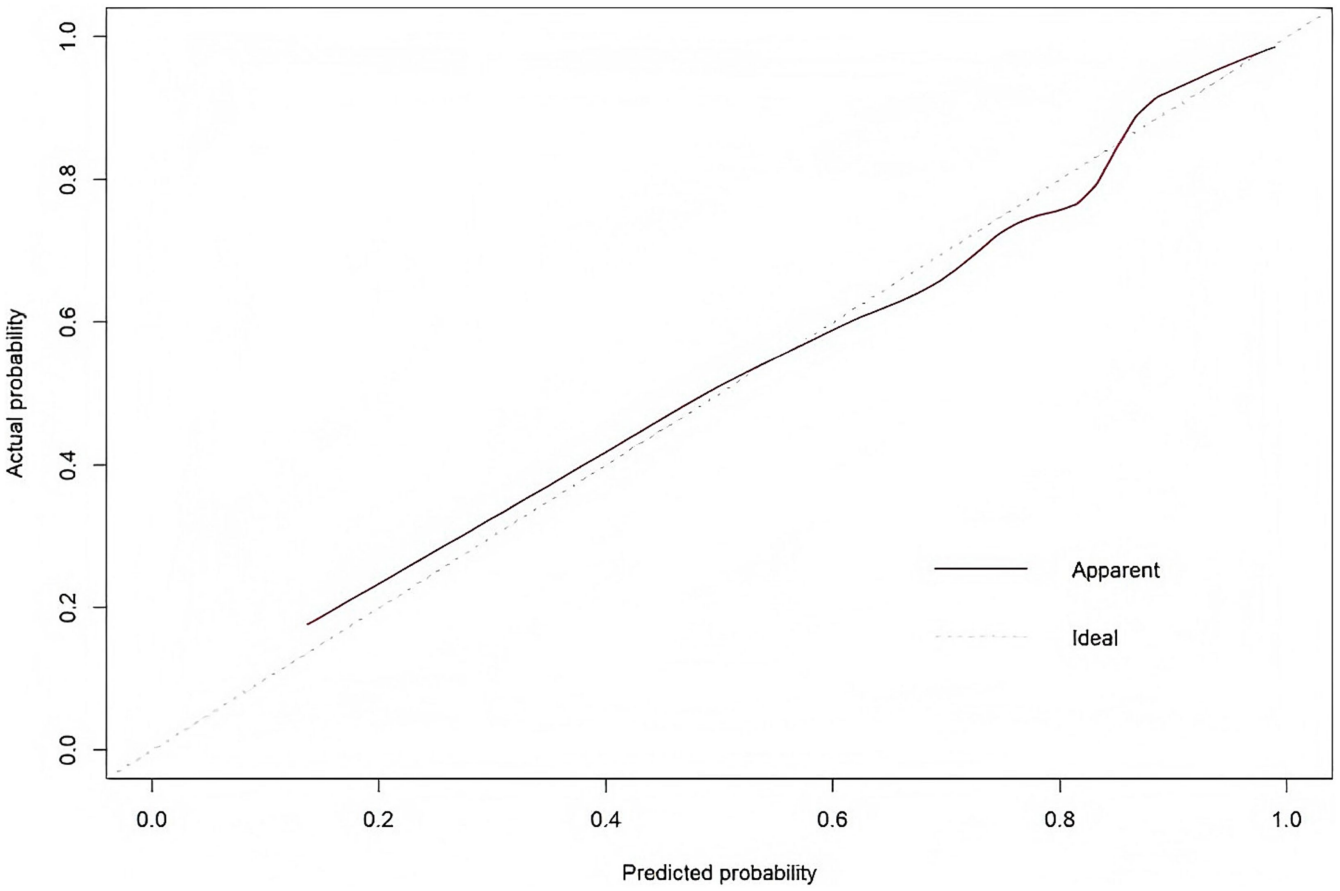
Calibration curve for nomogram-predicted prehospital delay risk in AIS patients.

Calibration curve: This curve primarily evaluates the calibration performance of the predictive model, measuring the consistency between the model’s “predicted probability of prehospital delay” and the “actual probability of delayed medical care occurrence.” The X-axis represents the probability predicted by the regression model for prehospital delay among AIS patients. In contrast, the Y-axis represents the actual probability of prehospital delay among AIS patients. The solid line (Apparent) closely aligns with the ideal 45° dashed line across the entire range of predicted probabilities, indicating strong agreement between the predicted and observed outcomes. The Hosmer–Lemeshow test yielded a *χ*^2^ value of 9.835 (*p* = 0.277), further confirming good model fit, as shown in Figure 3.

**Figure 4:**
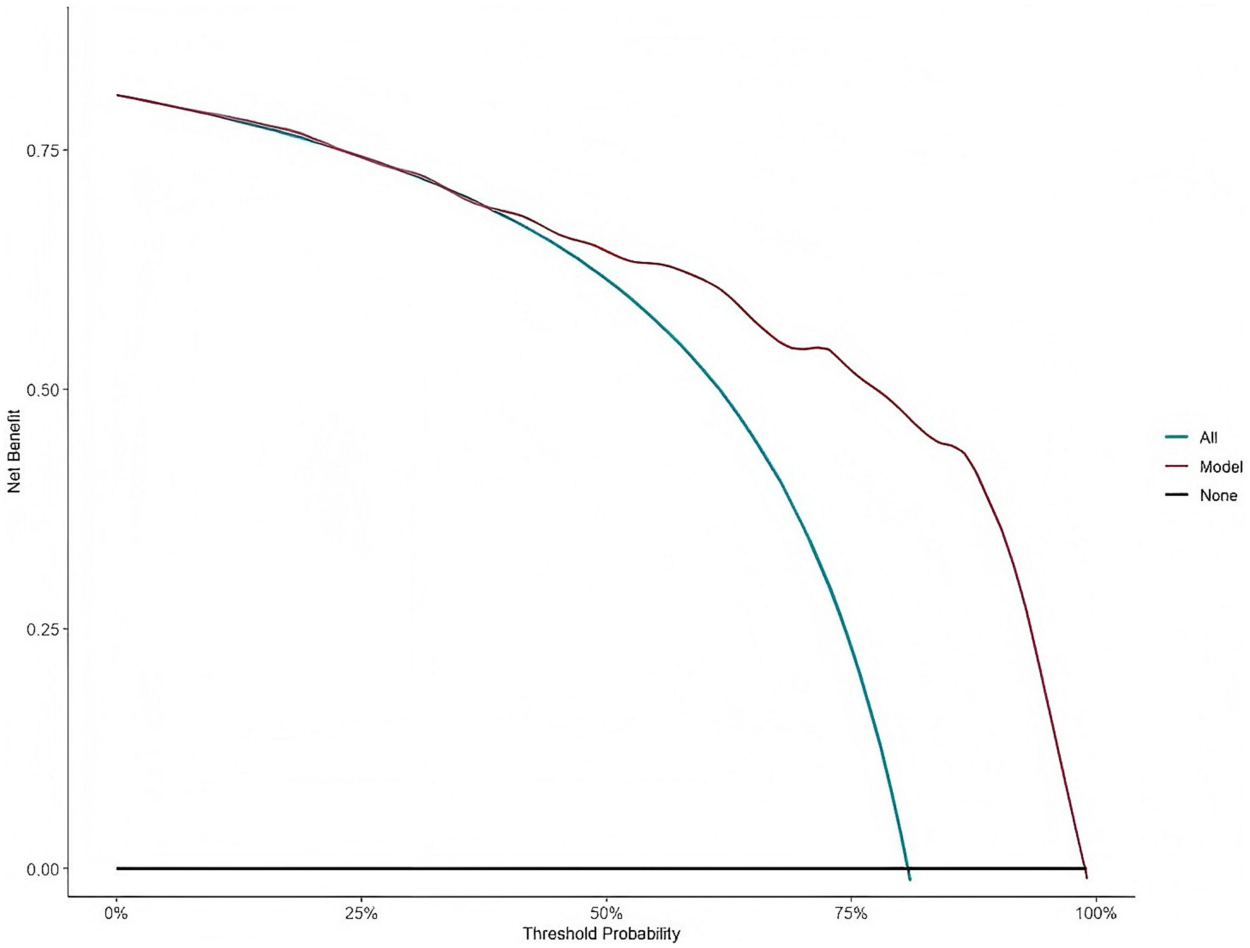
Decision curve analysis (DCA) for the prehospital delay risk forecast model in AIS patients.

The clinical utility of risk prediction models needs to be evaluated by conducting DCA to determine whether the model can deliver practical net benefits to clinical practice. DCA compares the constructed model curve with two extreme reference strategy curves (Figure 4): “All” (assuming that all AIS patients are at high risk of prehospital delay and implementing intervention) and “None” (assuming that no patients are at high risk and that no intervention is performed). The X-axis of the curve represents the risk threshold probability (the minimum probability of delayed medical care that clinicians consider worthy of intervention). In contrast, the Y-axis represents the net clinical benefit (balancing the benefits of correctly identifying high-risk patients against the potential harms of unnecessary interventions for low-risk patients).

In this study, the optimal cutoff value derived from the ROC curve was integrated to maximize the overall net clinical benefit. When the risk threshold was set at 0.625, 63 out of every 100 AIS patients could avoid prehospital delay via the predictive model. According to this decision curve, when the model threshold ranged from 0 to 75%, the curve consistently and persistently was above and to the right of both the “All” and “None” reference curves. These findings indicate that the constructed risk prediction model yields sustained positive net clinical benefits and has significant clinical utility.

## 4 Discussion

### 4.1 AIS is associated with a high incidence of prehospital delay

We enrolled 348 patients with AIS, of whom 281 had prehospital delay, resulting in a delay rate of 80.8%. This finding is comparable to the results of a multicenter study conducted in China, which reported a prehospital delay rate of 82.3% for AIS patients (30); this prehospital delay rate was higher than that (73.3%) reported in AIS patients in South India (31). This might be associated with disparities in regional healthcare resources, cultural characteristics of the population, study design, definitions of prehospital delay, and inclusion and exclusion criteria. A cross-sectional study conducted in Thailand reported a 70.0% delay rate within the first 3 h for patients with AIS (32). A multicenter study conducted in South Korea revealed that only 36.8% of patients with AIS reached the hospital within 4.5 h after symptom onset to undergo intravenous thrombolysis, leading to a delay in seeking medical care for 63.2% of patients (33). In the US, about 50% of AIS patients reach the hospital within 4.5 h, whereas in Spain, the prehospital delay rate for cases within 3 h is 48.7% (6). Although the rates of delayed medical care vary across different countries and regions, they remain consistently high overall. This situation highlights the urgent global need to prevent and control delayed care for AIS patients, with nations still facing significant challenges in this area. Despite an increase in public awareness of cerebrovascular diseases as societies develop, a lack of relevant health consciousness persists among the general population. As key agents in health education and emergency care, nursing staff play an irreplaceable role in this context. The findings of this study indicate a high rate of prehospital delay for AIS patients, suggesting that nurses need further training to strengthen targeted health education for patients with AIS. Therefore, healthcare authorities in all countries should gain a thorough understanding of the situation in their respective regions, collaborate with nursing staff, and develop prevention and control strategies tailored to regional characteristics to increase the level of care for AIS patients.

### 4.2 Evaluation of the predictive performance of a delay risk prediction model for AIS patients

The AIS delayed prehospital delay prediction model developed in this study demonstrated good predictive performance, featuring excellent discrimination and calibration. Clinicians can use this model as an objective and convenient risk stratification tool that can accurately classify patients with AIS. Generally, an AUC value between 0.7 and 0.9 indicates good discrimination. In this study, the area under the ROC curve was 0.87 (95% *CI*: 0.82–0.91), accompanied by a sensitivity of 0.79 and a specificity of 0.84, demonstrating robust predictive ability. The Hosmer–Lemeshow goodness-of-fit test resulted in a *p*-value of 0.277 (>0.05), which confirmed the high consistency between the predicted delay risk and the actual probability of occurrence. The calibration curve approached the ideal curve, indicating that the model had high accuracy. DCA revealed a positive net clinical benefit for patients using this predictive model, with thresholds ranging from 0 to 75%. The included predictors (level of education, history of cerebral infarction, Place of onset, Subsequent measures, and NIHSS score) are readily obtainable by nurses during clinical practice without requiring complex equipment or specialized tests. This highlights the need for clinical nurses to increase their vigilance toward such high-risk populations, calculate the probability of prehospital delay, and implement effective preventive and stratified interventions based on risk assessment. Thus, the risk prediction model developed in this study is practical and feasible, providing a reference for nursing professionals in other countries to assess high-risk populations for AIS.

### 4.3 Analysis of factors affecting prehospital delay among patients with acute ischemic stroke

#### 4.3.1 Patients with a lower level of education have a higher likelihood of experiencing prehospital delay

The results of this study showed that patients with an elementary school education or lower have a greater likelihood of prehospital delay, which agrees with the findings of Kim et al. (34). The reason lies in the inability of individuals with a low level of education to recognize early symptoms of AIS (35). They often misinterpret initial symptoms as fatigue or a common cold, leading to prehospital delay consultations and failing to recognize the critical time window, thereby compromising survival outcomes and quality of life. Therefore, for patients with a low level of education, healthcare providers should adopt personalized educational approaches. Besides regularly visiting communities to disseminate knowledge on AIS, they should collaborate with community workers to create educational resources such as community-based comic strips, short videos, and simulated drills for dialing 120 emergency services. This approach ensures that patients can master the “FAST” recognition method for stroke. Moreover, patients with a low level of education often rely on family members for medical decisions. We must also pay close attention to their family support systems. Training should extend beyond the patients to include in-home sessions with family members, providing them with knowledge about stroke health. This approach can significantly decrease the time spent prehospital delay (36).

#### 4.3.2 Effect of place of onset and subsequent measures on prehospital delay

The findings of this study highlight the clinical relevance of the location of onset to prehospital delay among patients with AIS. Although our logistic regression analysis did not achieve conventional statistical significance, many studies have reported that most AIS patients experience onset at home (37). Patients who experience onset at home are more likely to experience prehospital delay, which was also reported by Zhao et al. (38). Patients experiencing onset at home exhibit significantly greater rates of prehospital delay than those experiencing onset outdoors or in public places (39). The onset of an attack in most cases occurs during the night or when patients are asleep, and symptoms are discovered only upon awakening. This prevents quick identification of the condition and initiation of treatment. The onset of an attack at home often leads patients or caregivers to overlook atypical AIS symptoms, such as dizziness or limb numbness (40). Additionally, some patients are older adults living alone without relatives and are unable to call for help or use communication devices. Alternatively, symptoms may be severe at onset, preventing them from receiving emergency services. Therefore, interventions should expand beyond patients to encompass the entire family support system. The implementation of a “Family Stroke Emergency Plan” through role-playing drills allows family members to master emergency response knowledge. Our findings indicate that patients who first call 120 emergency services after the onset of symptoms are less likely to experience prehospital delay. However, only 7.5% of patients in the delayed group in this study initially dialed 120, which is significantly lower than that reported in South Korea (32.1%) (41). Most patients initially underestimate the severity of symptoms, often adopting a “wait-and-see” approach or prehospital delay until daylight. Emergency calls are mostly made only when symptoms worsen (e.g., impaired consciousness, hemiplegia, aphasia), leading to delayed treatment. Rural residents, facing distant medical facilities and feeling overwhelmed during onset, are more prone to entering a cycle of “observation and waiting.” This highlights the need for healthcare providers to screen high-risk populations proactively and implement systematic interventions (42). Public awareness videos should be played in high-traffic areas, such as residential elevator lobbies, plazas, shopping malls, and buses, to integrate knowledge regarding stroke into daily life. Moreover, family-focused health education regarding the prevention and treatment of AIS should highlight the significance of emergency medical services. By using diverse formats and channels, greater public awareness of stroke warning signs can be achieved, thereby reducing the chances of prehospital delay.

#### 4.3.3 Effect of a history of cerebral infarction on prehospital delay

A survey revealed that patients with AIS have a relatively high risk of prehospital delay. This may be attributed to differing symptoms compared to the initial AIS episode or because some patients who experienced minor strokes recovered well, potentially underestimating the severity of new symptoms (43). Some studies have identified a history of stroke as a major factor influencing the decisions of patients to prehospital delay. Such prior experiences can interfere with decision-making during a new episode, increasing the chances of delayed presentation. These findings suggest that clinical care providers should provide better crisis education for patients with cerebral infarction. Quarterly assessments of AIS recurrence risk and reinforcement of emergency knowledge should be conducted for patients and their families through outpatient follow-ups or internet-based nursing platforms (44). This ensures that they can make quick decisions when symptoms arise and seek medical attention promptly upon experiencing the symptoms of a stroke.

#### 4.3.4 Effect of the NIHSS scoring on prehospital delays

Patients with AIS who exhibit severe neurological deficits, such as impaired consciousness or aphasia, are more likely to experience prehospital delay. Without timely detection, these patients cannot seek hospital treatment promptly. High NIHSS scores serve as an independent risk factor for delayed in-hospital thrombolysis in AIS patients (45). Those with NIHSS scores ≥10 exhibit significantly greater rates of prehospital delays than those with NIHSS scores <10 (46). Therefore, nurses should immediately activate the “stroke rapid pathway” (e.g., prioritizing CT scans and preemptively contacting thrombolysis teams) and expedite triage for patients with NIHSS scores ≥10 (47). Targeted health education should be provided to patients with high neurological deficit scores, informing them and their families to call emergency services promptly upon the onset of symptoms. Additionally, regular follow-ups via outpatient clinics or online platforms should reassess the understanding of patients regarding the severity and urgency of a stroke, promptly correcting misconceptions. Through three-tiered “hospital-community-home” collaboration, community nurses should visit the homes of discharged high-NIHSS patients every month, reinforcing the principle of “seeking medical attention at the first sign of symptom change.” Nurses can also distribute weekly educational materials, such as videos or informational texts on “stroke severity and the urgency of emergency care,” via online platforms to gradually remove the misconception that “severe symptoms alone warrant medical attention.” This multidimensional intervention can effectively address the dilemma of high-NIHSS patients, who often struggle with limited self-rescue capabilities and insufficient family awareness.

## 5 Limitations

In this study, we established a predictive model for prehospital delay in patients with AIS by using influencing factors; the model has a good predictive value. It can assist clinicians in the early screening of high-risk populations for AIS, identifying vulnerable individuals, and implementing appropriate interventions to reduce adverse outcomes. However, this study had several limitations.

First, the definition of prehospital delay had limitations. In this study, “prehospital delay” was defined solely as prehospital delay (time from symptom onset to hospital arrival exceeding 4.5 h). While this definition holds clinical significance for patients eligible for intravenous thrombolysis, it excludes in-hospital delay factors, such as the time required for neuroimaging, neurological assessment, and thrombolysis decision-making. Therefore, the predictive model developed in this study primarily identifies risk factors for delayed patient presentation rather than the overall delay in initiating reperfusion therapy. This limitation may reduce the practical applicability of the model in clinical scenarios involving hospital-based processes. Future studies should consider more comprehensive factors, such as the precise “time from symptom onset to medication administration,” to more accurately reflect the entire process of prehospital delay from symptom onset to reperfusion therapy.

Second, the use of a convenience sampling method at a single tertiary center may have introduced selection bias. Our sample may not accurately represent the broader population of AIS patients, potentially underrepresenting those from diverse geographic or socioeconomic backgrounds or with severe strokes. This limits the generalizability of our findings, and this model should be applied with caution in other settings.

Third, the relatively small sample size precludes dividing the data into training and validation sets; validation is limited to internal methods rather than independent external datasets, a limitation that restricts the statistical power of the study. Therefore, future studies should incorporate more objective variables for external clinical validation to improve the performance and stability of the predictive model.

## 6 Conclusion

Our findings revealed that among 348 AIS patients recruited from a tertiary hospital in China, 80.8% experienced prehospital delay. We found that the level of education, history of cerebral infarction, place of onset, subsequent measures, and NIHSS were independent factors influencing prehospital delay for AIS. A risk prediction model developed based on these five factors demonstrated good calibration and discrimination. This model can help clinical nursing staff identify high-risk individuals early, allowing them to implement personalized interventions that reduce prehospital delay. This predictive model necessitates further clinical implementation. Nurses can deliver targeted, personalized interventions and optimize triage processes, thereby increasing the role of nursing care in optimizing time-sensitive treatment for AIS, reducing delays, and improving clinical outcomes.

## Data Availability

The original contributions presented in the study are included in the article/Supplementary material, further inquiries can be directed to the corresponding author.
