# Supplementary material for: Development and validation of a risk prediction model of prehospital delay in patients with acute ischemic stroke
Source: Front Public Health. 2026 Feb 11;14:1737563. doi: 10.3389/fpubh.2026.1737563 (PMC12932418; doi:10.3389/fpubh.2026.1737563)
Supplement: Supplementary file 1 [file Supplementary_file_1.docx]

Independent Variable Assignments

| Variable | Coding Method |
| --- | --- |
| Education | 1=Elementary school or lower, 2=Junior high school, 3=High school/vocational school, 4=Bachelor’s degree or higher |
| Place of Residence | 0=Rural, 1=Urban |
| Monthly Income | 1=Below ¥3,000, 2=¥3,001–4,000, 3=¥4,001–5,000, 4=Above ¥5,000 |
| History of Cerebral Infarction | 0=None, 1=Yes |
| History of atrial fibrillation | 0=No, 1=Yes |
| Place of onset | 1=Home, 2=Workplace, 3=Outdoors |
| Awareness of stroke onset after symptom onset | 0=Aware, 1=Unaware |
| Initial medical facility | 1=Community/Township Health Center, 2= County-level hospitals, 3=Tertiary General Hospitals |
| Subsequent measures | 1=Calling emergency services (120), 2=Immediately contact family or friends to go to the hospital, 3=Wait and monitor symptom changes |
| Transportation | 1=Ambulance, 2=Private vehicle, 3= Public Transportation, 4=Walking, 5=Taxi |
| Distance from initial hospital | 1=＜5km, 2=5-10km, 3=＞10km |
| NIHSS | Original value entered |
| PSSS | Original value entered |
| PBHSD-C | Original value entered |
